# Radiological assessment of cup anteversion with a novel 3D-printed highly-porous titanium dual mobility cup

**DOI:** 10.1186/s13018-025-05555-z

**Published:** 2025-02-08

**Authors:** Nicole Puteo, Edoardo Matteo Valentino, Vittorio Davidoni, Domenico Tigani

**Affiliations:** 1 Ospedale Maggiore Pizzardi, Bologna, Italy; 2 Istituto Ortopedico Rizzoli, Bologna, Italy

**Keywords:** Radiographic anteversion, Monobloc dual mobility, Titanium acetabular cup, Highly-porous titanium, Reliability, Accuracy

## Abstract

**Background:**

Cup anteversion after primary total hip arthroplasty (THA) with monobloc dual mobility cups (DMC) is difficult to measure on anterior-posterior (AP) pelvic radiographs because of the implant radiopacity and cup design which do not allow for an accurate visualization of the radiographic projection of the cup equatorial rim and the femoral head. This study aims to radiographically investigate on the reliability and accuracy of different measurement methods for cup anteversion in monobloc DMC THA, by comparing a novel titanium cup with conventional cup designs.

**Methods:**

97 THAs with a monobloc 3D-printed titanium DMC were radiographically assessed for cup radiographic anteversion (RA) one month postoperatively. RA were measured by three blinded observers through Lewinnek, Woo-Morrey methods and an open access mathematical software (GeoGebra), used as reference method. Intra- and interobserver reliability of RA measurements were evaluated for each method using intraclass correlation coefficient (ICC). Accuracy was assessed comparing Lewinnek and Woo-Morrey methods with GeoGebra. Moreover, further 98 THAs with conventional different brand DMC were radiographically assessed as control group by using the same methods.

**Results:**

ICC for intra- and interobserver reliability for RA measurements with GeoGebra, Lewinnek and Woo-Morrey methods were 0.975–0.980, 0.978 − 0.965, and 0.979 − 0.958, respectively, for the titanium DMC group. Lewinnek resulted more accurate for RA than Woo-Morrey, differing by + 0.4° (*p* = 0.06) and + 4.4° (*p* < 0.001) from GeoGebra, respectively. ICC for intra- and interobserver reliability for GeoGebra, Lewinnek and Woo-Morrey methods were 0.848 − 0.756, 0.843 − 0.801, and 0.965 − 0.958, respectively, for the control DMC group. Lewinnek and Woo-Morrey methods differed by -2.3° and + 5.1° from GeoGebra, respectively (*p* < 0.001).

**Conclusions:**

RA measurements are more consistent, repeatable and accurate with a titanium DMC than standard DMC, due to the minor radiopacity of the former cup which enable RA measurements on AP radiographs. Conversely, RA measurements of conventional DMC are more consistent but less accurate if performed on cross-table lateral radiographs by Woo-Morrey method than AP radiographs.

## Introduction

The orientation of the acetabular cup after total hip arthroplasty (THA) is an important radiographic parameter to assess as it affects hip stability, femoral impingement, bearing wear and implant longevity. While cup inclination is easily measured on anterior-posterior (AP) pelvis radiographs, cup anteversion is not so straightforward to measure.

The acetabular anteversion is historically defined as anatomical, radiographic, and operative according to which plane the angle between the projection of the acetabular axis passing through the center of the socket and the perpendicular coronal plane is measured on [1]. Anatomical, or true, anteversion is defined as the angle between the transverse axis and the acetabular axis when this is projected on to the transverse plane. Radiographic, or planar, anteversion (RA) is defined as the angle between the acetabular axis and the coronal plane [1]. While anatomical anteversion is easily and accurately measurable on transverse plane views visible through computed tomographic (CT) scans of the pelvis [2], several methods have been previously described and investigated in THA with standard acetabular components to measure cup RA through plain radiographic views including AP and cross-table lateral of the hip [3–9]. Historically, anteversion assessment was not so frequently reported in studies concerning dual mobility THA. Although inclination can be easily measured on plain AP pelvis radiographs, in case of monobloc dual mobility cup (DMC), RA measurement is more challenging and could lead to inaccurate values, because of the radiopacity of cobalt-chromium-molybdenum (CoCr) or stainless steel (SS), which are the conventional manufacturing metal alloys for such components, and the component designs, which do not allow a clear visualization of the elliptical radiographic projection of the equatorial rim and the femoral head profile [10].

To measure cup anteversion of conventional monobloc DMC, the method by Woo-Morrey on cross-table lateral hip radiograph is one of the most cited [8, 11, 12]. However, this method, due to patient positioning during cross-table lateral radiograph, has showed low precision with high variability and measurements that often exceeded more than 10° [13].

A monobloc DMC made with titanium alloy, thanks to its higher radiotrasparency than CoCr and SS, should allow a better visualization of the elliptical projection of the cup rim on standard AP radiographs. Therefore, our hypothesis was that a novel off-the-shelf titanium monobloc DMC should theoretically allow a more accurate and reliable measurement of cup RA on AP pelvic radiograph than conventional monobloc CoCr or SS DMCs.

The present retrospective study aims to investigate the reliability and accuracy of different methods for cup RA measurement with a novel titanium monobloc DMC in comparison to conventional DMC designs used in primary THA.

## Methods

### Study procedure

All primary THAs performed at our institution with monobloc DMC from January 2012 to December 2023 have been identified through hospital database search. In January 2022 a novel off-the-shelf titanium monobloc DMC was introduced in our clinical practice.

Inclusion criteria were patients undergoing primary THA for all diagnosis with cementless monobloc titanium, CoCr, or SS DMC of different brands with available radiographic follow-up taken postoperatively at 1-month follow-up with AP pelvic and hip cross-table lateral views. Exclusion criteria were patient with significant spinal deformities, or stabilization, or arthrodesis of the lumbar spine and poor quality radiographic images.

Eligible patients who met all inclusion-exclusion criteria were enrolled in this retrospective, observational, comparative study. 97 hips which received the same monobloc titanium DMC from the same manufacturer were included as titanium group, while 98 hips which received conventional CoCr or SS monobloc DMC from different manufactures were included as control group.

### Titanium DMC

**Fig. 1:**
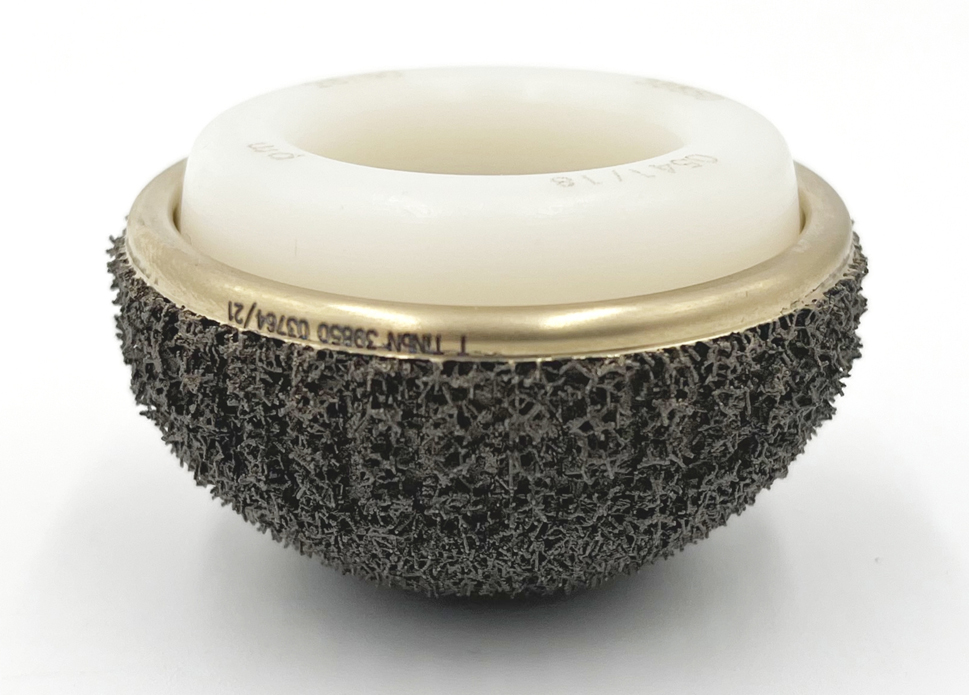
The investigated titanium DMC, the Acorn Traser Dual Mobility cup (Permedica Orthopaedics, Merate, Italy). The cup is 3D-printed with selective laser-melting using Ti6Al4V powder in one single process without continuity solution between the bulky portion and the outer highly-porous portion, without material inner discontinuity. The highly-porous structure (Traser^®^) is characterized by an open, fully-interconnected, irregular porosity (mean pore size 520 μm, porosity 70%)

The titanium DMC is a novel, off-the-shelf, cementless, highly-porous, titanium monobloc DMC, the Acorn Traser^®^ cup (Permedica Orthopaedics, Merate, Italy). The cup is fully 3D-printed with titanium alloy for powder bed fusion. Its design has a polar flattened hemispherical profile with a flat cup opening plane. The articular surface is coated with titanium-niobium nitride to allow articulation against the polyethylene mobile head (Fig. 1).

### Control DMC

Three brands of cementless CoCr or SS monobloc DMC from different manufactures were grouped as control group: Acorn (Permedica, Merate, Italy), Quattro™ (Groupe Lépine, Genay, France), and Novae^®^ Sunfit (Serf, Décines-Charpieu, France).

Acorn cup is a forged SS DMC with polar flattened hemispherical shape, a tilted cup opening plane, and equatorial grooves. Quattro™ cup is a CoCr DMC with hemispherical shape, “hat” design, and equatorial fins. Novae Sunfit is a forged SS DMC with hemispherical shape with additional 3 mm cylindrical portion to form a cylinder-spherical shape, a flat cup opening plane, and equatorial rib pattern. Implant fixation was obtained in all three brands by primary press-fit and secondary osseointegration through plasma-sprayed by titanium-hydroxyapatite bilayer coating.

### Radiographic measurements

All available radiographs taken at 1-month follow-up were searched for each enrolled patient on the hospital picture archiving and communication system and assessed for RA measurements according to the following methods for all hips of titanium and control groups.

**Fig. 2:**
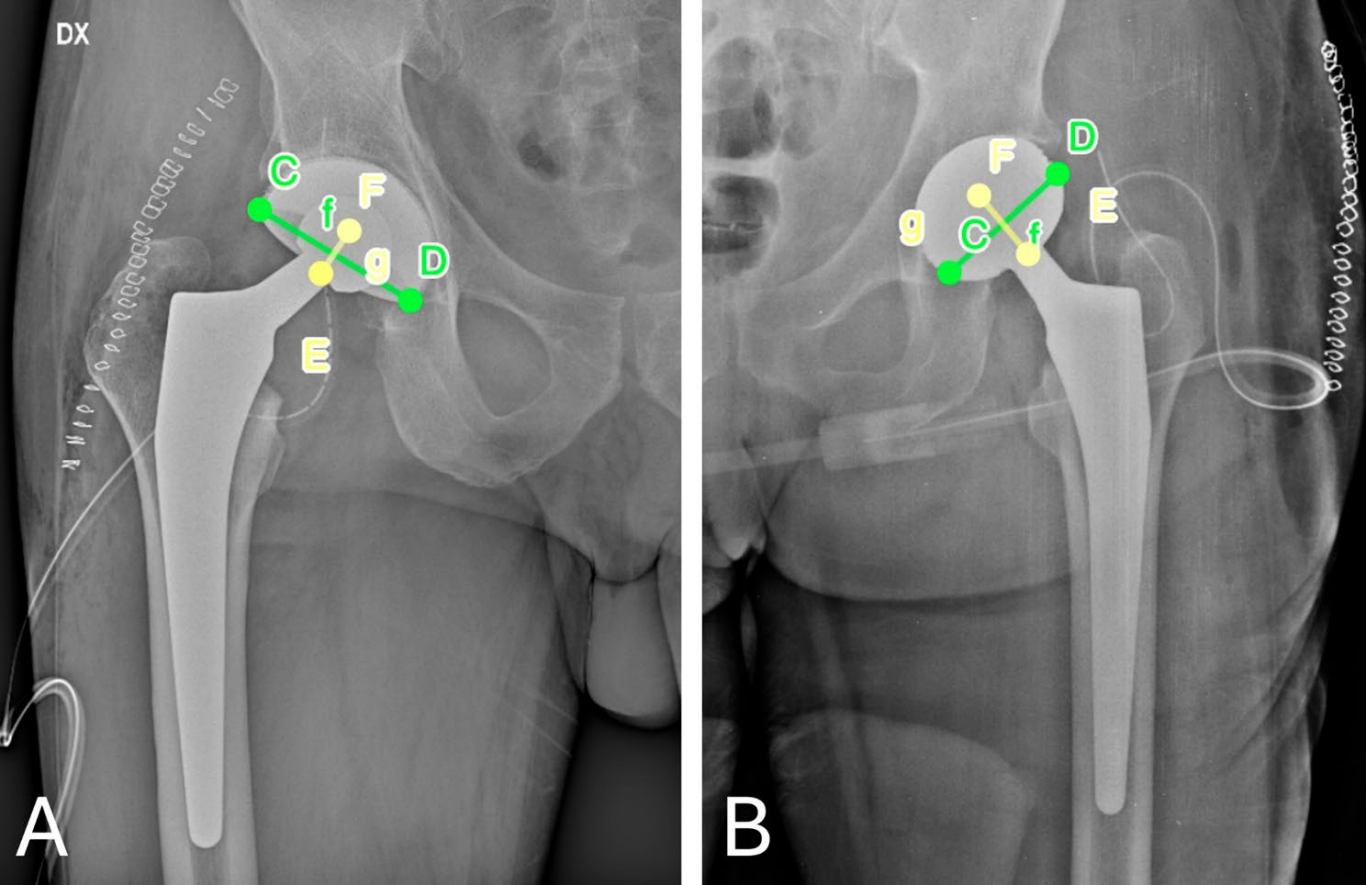
Illustration of the method of Lewinnek et al. **A**: Anterior-posterior postoperative radiograph of a hip in the titanium group with the cup anteversion method according to Lewinnek et al. The long (D2 = f) and short (D1 = g) axes of the visible projected ellipse on plain AP hip radiograph are easily drawn and their measures are used to calculate the cup radiographic anteversion. **B**: Anterior-posterior postoperative radiograph of a hip in the control group with the cup anteversion method according to Lewinnek et al. The projected ellipse of the cup is not well visible, since the medial ellipse profile is hidden by the cup and thus, the short (D1 = g) axis of the ellipse is approximately drawn. E, F extremity points of the long axis. C, D extremity points of the short axis

The measurement of the RA of the acetabular component was performed on AP pevic radiograph with the method described of Lewinnek et al. [3] using the known following formula:

$$\:Version\hspace{0.17em}=\hspace{0.17em}arcsin\:(D1/D2)$$

where D1 is the distance of the short axis of the ellipse and D2 is the long axis of the acetabular component, reflecting the maximum diameter of the implant (Fig. 2).

**Fig. 3:**
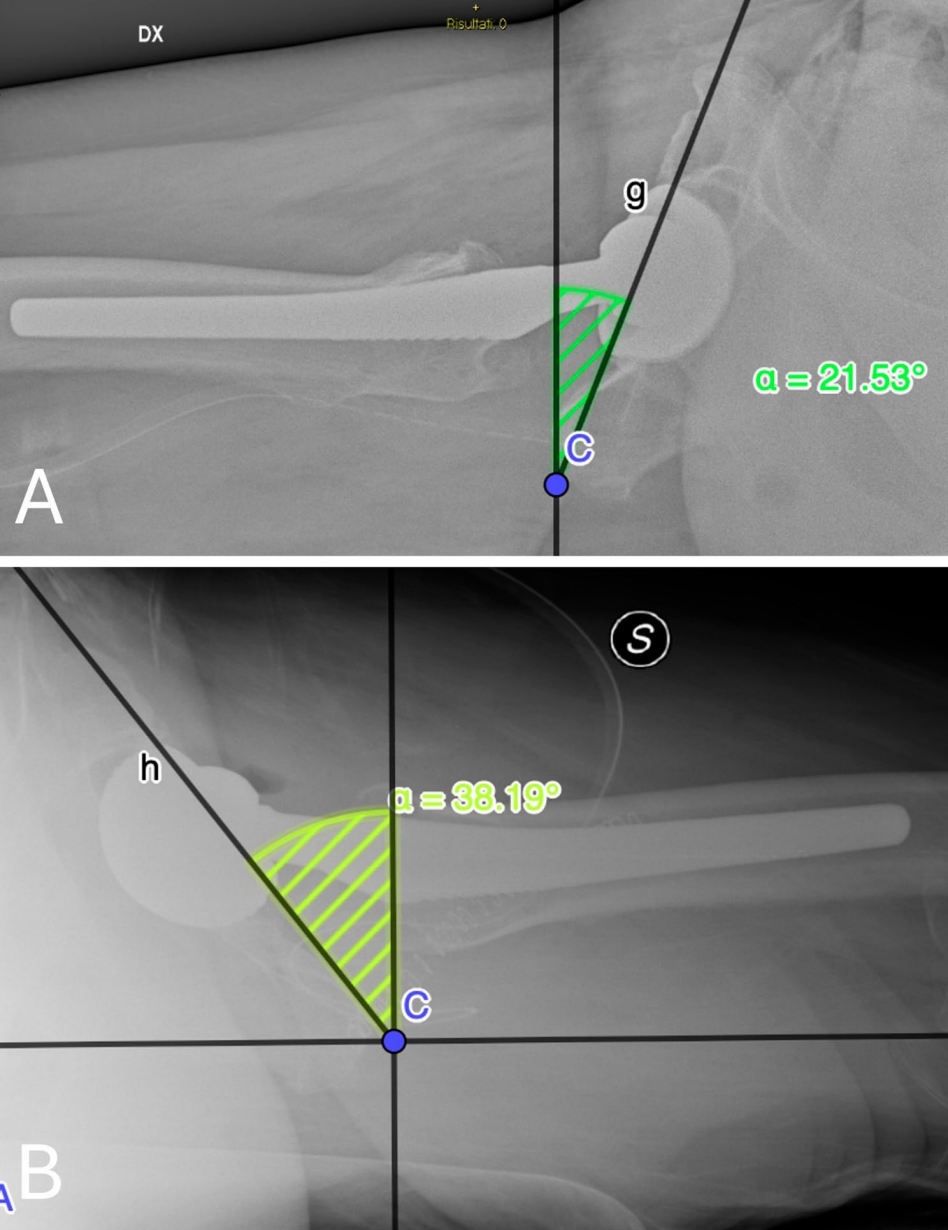
Illustration of the method of Woo and Morrey at al. The cup radiographic anteversion is the angle between the line tangent to the cup opening and the line perpendicular to the ground measured on cross-table lateral radiographic view of the hip. **A**: Cross-table lateral postoperative radiograph of a hip in the titanium group with the cup anteversion method according to Woo and Morrey et al. **B**: Cross-table lateral postoperative radiograph of a hip in the control group with the cup anteversion method according to Woo and Morrey et al

Measurement of the RA of the acetabular component was performed according to the method described by Woo and Morrey [8]. Unlike Lewinnek’s method which uses AP plain radiographs, version is not calculated using a formula but rather it is directly measured on cross-table lateral radiographs, on which anteversion and retroversion can be distinguished [13]. Version is directly calculated as the angle between the line touching the opening rim of the acetabular component and a line perpendicularly drawn to the table (Fig. 3).

Measurement of the RA of the acetabular component was then performed using the GeoGebra software, which was established as reference method in the present study [14]. GeoGebra is an open access mathematics software which allows to automatically calculate the RA of the acetabular cup through a validated method described by Bachhal et al. [15, 16].

GeoGebra is able to draw an ellipse by manually setting three points which identify the two ellipse foci and one point on the circumference on an image (the uploaded digital AP radiograph). This ellipse can be moved to best fit the outline of the elliptical projection of the face of the acetabular cup. The centre of the ellipse, circular outline of the acetabular cup as well as its major and minor axis can be similarly drawn.

**Fig. 4:**
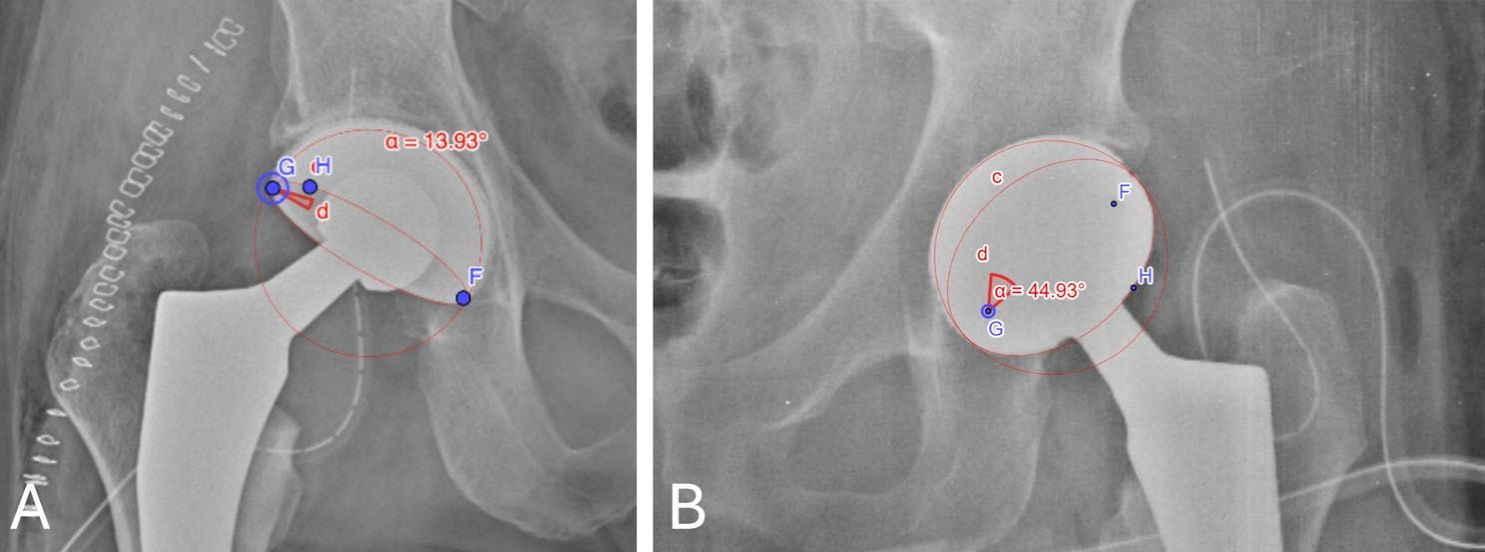
Illustration of the method with GeoGebra software. The points G and F correspond to the ellipse foci. The point H is a point along the elliptic profile. The software allows to move the two foci in order to obtaining perfect matching between the drawn ellipse and the elliptic projection of the cup opening rim. The cup radiographic anteversion (a) is automatically calculated by the software. **A**: Anterior-posterior postoperative radiograph of a hip in the titanium group with the cup anteversion measured with GeoGebra tool. **B**: Anterior-posterior postoperative radiograph of a hip in the control group with the cup anteversion measured with GeoGebra tool

RA angle of the acetabular cup is automatically calculated by the programme, as the angle subtended by minor semi-axis at either focus of the elliptical projection of the cup rim, as base and hypotenuse representing the semi-minor and semi-major axis, respectively, as easily demonstrated by a geometrical formula (Fig. 4) [16]. This value was regarded as the reference standard for the measurement of the RA of the acetabular component in the present study.

### Assessment of reliability and accuracy

Reliability can be defined as consistency in measurements. The GeoGebra and Lewinnek methods using AP plain radiographs and the Woo-Morrey method using cross-table lateral radiographs were performed independently and at different times by three observers (N.P, E.M.V., and V.D.), using the same protocol. The intra-observer reliability for each method was evaluated by one observer (N.P.) and was reassessed twice at intervals of 2 weeks for the images of all 97 + 98 patients [17]. The inter-observer reliability across the three observers was calculated for each method. All measurements were calculated with the observer blind to the patient information, implant brand, and other observers’ values [18].

Accuracy was defined as the proximity to the GeoGebra software value (the reference standard) and was calculated by comparing the mean values from Lewinnek and Woo-Morrey methods to the mean value obtained using the GeoGebra programme.

### Statistical analysis

Intra- and inter-observer reliability were evaluated using intraclass correlation coefficients (ICCs) with 95% confidence intervals (CIs), with ICC values less than 0.5 are suggestive for poor reliability, values between 0.5 and 0.75 indicate moderate reliability, values between 0.75 and 0.9 indicate good reliability, and values greater than 0.90 indicate excellent reliability [19]. Normality distribution was tested with Shapiro-Wilk test. 2-sided Student t-test or Wilkoxon signed-rank paired t test were used when distribution was found normal or not, respectively, to evaluate the accuracy of each method compared to GeoGebra programme. To find a significant difference (*p* < 0.05) of 5° with 10° standard deviation (SD) in mean cup anteversion measurement between different methods, we performed a priori sample size analysis by using a superiority t-test with power of 80% and alpha of 0.05, which gave 63 cases per method. Statistical analyses were conducted using the free open source statistical software JASP version 0.18.3 (Amsterdam, The Netherlands), and the statistical significance was set at *p* < 0.05.

## Results

Patient demographic data of the included hips were reported in Table 1.

**Table 1:** Demographic data of the enrolled patients

	Titanium group	Control group
N. of hips	97	98
Brand	Acorn Traser DMC	Acorn Quattro Sunfit
N. of female; male	64; 33	57; 41
Mean age at operation ± SD	76.3 ± 4.5	73.8 ± 3.2
Diagnosis		
Osteoarthrosis	21	15
Femoral neck fracture	76	83

In the titanium DMC group, ICCs for intraobserver reliability of RA measurements with GeoGebra, Lewinnek, and Woo-Morrey methods were 0.975, 0.978, and 0.979, respectively, while ICCs for interobserver reliability were 0.980, 0.965, and 0.958, respectively (Table 2). Lewinnek resulted more accurate than Woo-Morrey in RA measure, differing by mean + 0.4° (SD 2.1) (*p* = 0.06) and + 4.4° (SD 5.9) (*p* < 0.001) from GeoGebra, respectively (Table 3).

**Table 2:** Titanium group. ICC for intraobserver and interobserver reliability for GeoGebra, Lewinnek, and woo-Morrey RA measurement methods. ICC, intraclass correlation coefficient. CI, confidence interval

	GeoGebra	Lewinnek	Woo-Morrey
ICC for Intraobserver reliability (95% CI)	0.975 (0.966–0.983)	0.978 (0.969–0.985)	0.979 (0.971–0.985)
ICC for Interobserver reliability (95% CI)	0.980 (0.972–0.986)	0.965 (0.952–0.976)	0.958 (0.941–0.971)

**Table 3:** Titanium group. Accuracy for Lewinnek and Woo-Morrey RA measurement methods using GeoGebra as reference method. SD, standard deviation

	GeoGebra	Lewinnek	Woo-Morrey
Mean RA (SD)	19.7° (8.4°)	20.1° (8.6°)	24.2° (10.1)
P value		0.063	< 0.001

In the control DMC group, ICCs for intraobserver reliability for GeoGebra, Lewinnek, and Woo-Morrey methods were 0.848, 0.843, and 0.965, respectively, while ICCs for interobserver reliability were 0.756, 0.801, and 0.958, respectively (Table 4). Lewinnek and Woo-Morrey significantly differed by mean − 2.3° (SD 4.9) and + 5.1° (SD 6.8) from GeoGebra (*p* < 0.001), respectively (Tables 5 and 6).

**Table 4:** Control group. ICC for intraobserver and interobserver reliability for GeoGebra, Lewinnek, and woo-Morrey RA measurement methods. ICC, intraclass correlation coefficient. CI, confidence interval

	GeoGebra	Lewinnek	Woo-Morrey
ICC for Intraobserver reliability (95% CI)	0.848 (0.795–0.890)	0.843 (0.789–0.887)	0.965 (0.952–0.976)
ICC for Interobserver reliability (95% CI)	0.756 (0.667–0.825)	0.801 (0.736–0.855)	0.958 (0.942–0.970)

**Table 5:** Control group. Accuracy for Lewinnek and Woo-Morrey RA measurements using GeoGebra as reference method. SD, standard deviation

	GeoGebra	Lewinnek	Woo-Morrey
Mean (SD)	20.3° (7.9°)	18.0° (6.0°)	25.5° (11.12°)
P value		< 0.001	< 0.001

**Table 6:** Accuracy comparison between Titanium vs. Control groups of Lewinnek and Woo-Morrey methods using Geogebra reference. SD, standard deviation

	Titanium group	Control group	*P* value
Mean (SD) RA angle difference (Lewinnek– GeoGebra)	+ 0.4° (2.1)	-2.3° (4.9)	< 0.001
Mean (SD) RA angle difference (Woo-Morrey– GeoGebra)	+ 4.4° (5.9)	+ 5.1° (6.8)	0.521

Lewinnek accuracy is significantly higher in the titanium group than the control group (*p* < 0.001), while Woo-Morrey accuracy is low and not different in both titanium and control group (*p* = 0.5).

## Discussion

The present study investigated on acetabular cup orientation, in particular on reliability and accuracy of cup RA through radiographic measurements after primary THA, comparing a novel monobloc titanium 3D-printed DMC with other conventional monobloc DMCs. To the best of our knowledge, this study represents the first investigation on accuracy and reliability of RA measurements specific to monobloc DMC in THA. In literature no similar study was found specifically with DMC.

The findings of this study support the hypothesis that a titanium monobloc DMC might allow more accurate and reliable RA measurements on AP radiographs than conventional monobloc CoCr or SS DMC designs could do. As a matter of fact, Titanium alloy, having a density that is approximately half the density of CoCr or SS, features less radiopacity to x-rays than CoCr and SS alloys, as it is well recognizable when looking at standard radiographs after THAs with cementless metal-backed titanium acetabular shells coupled with polyethylene liners, in which usually it is possible to easily identify radiographic projections of the femoral head, equatorial cup rim or cup holes. On the other hand, when looking at radiographs of hip hemiarthroplasties with bipolar heads, THAs with large head metal-on-metal implants, or conventional monobloc DMCs, it is difficult, or even impossible sometimes, to detect projections of the femoral head profile or the anterior portion of the component rim due to the higher radiopacity of CrCo and SS.

Therefore, this novel cementless titanium DMC allows a clear visualization of the radiographic projections of the cup opening rim and the articular femoral head on AP radiographs similarly to AP radiographs of standard single mobility cementless metal-backed titanium acetabular cups.

Within the most established methods for cup anteversion measurement, it was decided to use for this research those described by Lewinnek et al. and Woo-Moorey et al. and also to compare RA results found from these methods with RA results found from the method described by Bachhal et al. used as referecence values [3, 8, 15, 16]. The reasons for this choice are explained herebelow in details.

The former method was selected because of its excellent reliability and its equivalence with Liaw et al. formula, as it can be easily demonstrated through few trigonometric Eqs. [7, 20]. Moreover, Lewinnek method, requiring only the long and short ellipse axes measurements and one simple formula to be calculated, is easier and faster than other described methods [4–6]. Widmer et al. method is based on an approximately linear relationship between radiographic cup anteversion from 10° to 30° and S/TL ratio, where S is the projected ellipse minor axis and TL the total length of the minor axis extended to the cup polar apex [4]. Due to the polar flattening of the titanium DMC investigated in this research and the different cup designs included in the control group, Widmer et al. method was not considered for this purpose, since it is best applicable for fully hemispheric cups.

Woo-Moorey method was chosen because of its excellent reliability, cup retroversion is detectable from anteversion and because it is easy and quick requiring only one angle measurement in the cross-table lateral view of the hip, which corresponds to the RA [8, 20]. Interestingly, in this radiographic view, the radiographic cassette is positioned at 35–45° from the patient cranio-caudal axis and perpendicular to the coronal plane, therefore, the plane of the cassette is approximately parallel to the plane where the axis of the acetabulum lies and where the acetabular RA is defined according to Murray et al. [1]. Obviously, acetabular RA taken on cross-table lateral view is affected by cassette positioning and patient positioning in supine position when the acetabulum orientation can change due to pelvic tilting or rotation.

There is another acetabular anteversion method based on cross-table lateral view which has been described to be independent from pelvic tilt and patient’s positioning: the ischiolateral method [21]. The ischiolateral method uses the long axis of the ischial tuberosity as bony landmark, and it was reported to be more accurate than Woo and Morrey’s method, which instead uses film ground as reference [22]. However, the ischiolateral method overestimates the acetabular RA by adding an angle equal to the angle between the horizontal film edge and the long axis of the ischial tuberosity [22]. Thus, the Woo-Morrey remained the method of choice.

For cup RA measurement reference it was decided to use an open-access mathematics software, GeoGebra, which allows drawing and matching an ellipse with two foci and one point on the same ellipse with the outline of elliptical projection of the face of the acetabular cup on an AP radiographic image of the hip imported into the software. This new simplified method has been validated and it showed high reliability and accuracy [15, 16].

In our study we have found excellent intra- and inter-observer reliability in titanium group for each considered method (ICC > 0.9). In titanium group Lewinnek method has revealed high accuracy with a non significant difference of + 0.4° from GeoGebra method. Conversely, Woo-Morrey method has showed significantly lower accuracy, exceeding by + 4.4° cup RA from GeoGebra method.

In the control group it was found good intra- and inter-observer reliability for GeoGebra and Lewinnek methods (0.75 < ICC < 0.9), but excellent intra- and inter-observer reliability for Woo-Morrey. These results confirm how RA measurements of radiopaque acetabular components, as standard monobloc DMCs, have low reliability on plain AP pelvic radiographs but higher reliability if measured on cross-table lateral view. However, both Lewinnek and Woo-Morrey methods demonstrated to be less accurate with significant differences from GeoGebra in the control group. Therefore, when assessing cup anteversion with a conventional DMC, even using the Woo-Morrey method due to its simplicity and reliability, the observer will incur a measurement error of at least 4–5 degrees from the real value.

The results in titanium group are consistent with the literature on RA measurements with standard acetabular cups. Similar findings were reported in the research by Park et al., who showed excellent reliability for all cup anteversion methods in comparison to PolyWare^®^ programme used as reference, with the highest ICC values with Woo-Morrey [20]. Park et al. found significantly lower accuracy for Woo-Morrey with approximately + 8° of mean cup anteversion difference from the reference, consistently with our findings. Nevertheless, Park et al. found significant lower accuracy also for Lewinnek but not for Liaw et al. method, which as said, are based on basically equivalent formulae [20]. Similar results were found by Nomura et al. who reported an overestimation of + 4° averagely using Woo-Morrey method in comparison to CT anteversion measurements [23].

Other studies found that measurements on cross-table lateral view overestimate acetabular anteversion more than 5° averagely in comparison to AP radiographic methods [20, 24, 25].

Proper positioning of the acetabular component is a key factor for successful THA [26, 27]. It has been recently showed how often the acetabular cup orientation after THA according to the historical Lewinnek safe zone does not properly match the acetabular cup position suggested by the patient’s specific functional planning when considering patient specific spino-pelvic parameters [28].

It is well recognized that a DMC is much more forgiving for cup malpositioning than a conventional unipolar acetabular shell with a fixed liner. Monobloc DMCs, having a higher jump distance than single-articulation cups, reduce the risk of head dislocation in case of malpositioning or spinopelvic disorder that lead to acetabular orientation functional changes [29]. Anyway, malposition of DMCs may lead to undesiderable side effects. Within DMC failures, cup malposition can be one of the initial reasons for revision for iliopsoas impingement, dislocation [30, 31]. A DMC placed with a poor anteversion or even retroverted could cause anterior impingement with femoral neck with metal indentation and wear of the acetabular rim with subsequent metallosis and metal ion release [32, 33]. Thus, the possibility of an easily and reliable radiographic assessment for DMC orientation is advisable, even after revision THA and acute THA for femoral neck fracture when cup malpositioning can occur more frequently causing instability [34, 35]. Cup malposition can also contribute to dislocation or intraprosthetic dislocation of the mobile polyethylene head of modular DMC [36].

In authors opinion the findings of this study have mainly a research importance rather than clinical applications since an error of + 4.4° or more of cup anteversion measurement, rather than a more accurate measurement, has nothing to deal with the actual intraoperative cup placement and its clinical implications for the patient. Therefore, the findings of this study may suggest how a more accurate cup anteversion assessment may lead to a more correct cup malpositioning diagnosis, even in case of a DMC THA.

Strengths of the present study were the sample size of the study population which enable for power and statistical significance of the results, the control group which included different DMC designs and the novelty of a RA investigation with monobloc DMC which was never specifically investigated before.

The present study has also some limitations. This study is about measurements performed on retrospective radiographic follow-ups of a retrospective cohort. Therefore, all assessed radiographs were taken at different time points during a long time period without a standardized protocol for patient positioning, so that a certain variability in radiographic projections during radiographic follow-ups was expected.

Second, it was decided to include in the control group different brands of monobloc DMC which are all manufactured with CoCr or SS alloys, in order to have a comparison group which could be not design-specific. However, the DMCs included in the control group basically differ in their equatorial portion designs. Probably, not symmetrical different cup opening designs could have biased correct RA measurements.

Third, GeoGebra software is a new open access tool for cup anteversion measurements which we had used as reference method. Even if this software is based on a validated method with standard press-fit or cemented hemispherical cup, this method has never been investigated before with standard DMC. Moreover, GeoGebra software imports only plain radiographic images but does not import also 3D-models of acetabular component brands, conversely to other specific radiographic analysis software as PolyWare^®^ or EBRA-Cup™. Then, GeoGebra could be less precise and accurate in comparison of other best-known imaging softwares.

## Conclusions

Acetabular cup RA measurements after primary THA are more consistent, repeatable and accurate with a titanium DMC than conventional CoCr or SS DMCs, due to minor radiopacity of the former which enable RA measurements on AP radiographs. Conversely, RA measurements of conventional DMC are more consistent but less accurate if performed on cross-table lateral radiographs than AP radiographs.

## Data Availability

Data generated and analysed during the current study are available upon reasonable request to the corresponding author.

## Abbreviations

THA: Total hip arthroplasty
DMC: Dual mobility cup
CoCr: Cobalt-chromium-molybdenum
SS: Stainless steel
AP: Anterior-Posterior
RA: Radiographic anteversion
ICC: Intraclass correlation coefficient
SD: Standard deviation
CI: Confidence interval
